# Do Development and Diet Determine the Degree of Cannibalism in Insects? To Eat or Not to Eat Conspecifics

**DOI:** 10.3390/insects11040242

**Published:** 2020-04-14

**Authors:** Francisco J. Fernandez, Manuel Gamez, Jozsef Garay, Tomas Cabello

**Affiliations:** 1 Center for Research in Mediterranean Intensive Agrosystems and Agrifood Bioechnology (CIAMBITAL), Agrifood Campus of International Excellence (CEIA3), University of Almeria, Ctra. Sacramento, s/n, 04120 La Cañada, Spain; javierfermal@gmail.com (F.J.F.); mgamez@ual.es (M.G.); 2 Ecology and Theoretical Biology, Eötvös Loránd University, Pázmány Péter sétány1/c, 1117 Budapest, Hungary; garayj@caesar.elte.hu

**Keywords:** *Nabis pseudoferus*, *Nesidiocoris tenuis*, predatory insect, generalist predator, true omnivore, intraguild predation, ontogeny, biological control, mathematical model

## Abstract

Cannibalism in insects plays an important role in ecological relationships. Nonetheless, it has not been studied as extensively as in other arthropods groups (e.g., Arachnida). From a theoretical point of view, cannibalism has an impact on the development of more realistic stage-structure mathematical models. Additionally, it has a practical application for biological pest control, both in mass-rearing and out in the field through inoculative releases. In this paper, the cannibalistic behavior of two species of predatory bugs was studied under laboratory conditions—one of them a generalist predator (strictly carnivorous), *Nabis pseudoferus*, and the other a true omnivore (zoophytophagous), *Nesidiocoris tenuis*—and compared with the intraguild predation (IGP) behavior. The results showed that cannibalism in *N. pseudoferus* was prevalent in all the developmental stages studied, whereas in *N. tenuis*, cannibalism was rarely observed, and it was restricted mainly to the first three nymphal stages. Cannibalism and intraguild predation had no linear relationship with the different cannibal–prey size ratios, as evaluated by the mortality rates and survival times, although there were variations in cannibalism between stages, especially for *N. pseudoferus*. The mathematical model’s implications are presented and discussed.

## 1. Introduction

Omnivores can be classified according to their diet or their role in ecological food webs [1]. Omnivory may be opportunistic, obligatory or facultative, based on the relative importance of plant and prey materials in the insect’s diet. However, according to their ecological role in food webs, an omnivore that feeds on more than one trophic level is commonly termed a “trophic omnivore” [1]. Intraguild predation is an example of trophic omnivory in which a predator consumes other predators with whom it shares a common herbivore prey [1,2]. “True omnivory”, therefore, is a particular case of trophic omnivory in which the consumer feeds on both plants and prey [1]. According to Hurd [3], generalist arthropod predators are typically bitrophic: they simultaneously occupy the third and fourth trophic levels by virtue of feeding both on herbivores and each other, i.e., they engage in intraguild predation (IGP).

At the same time, predation can be either between species or among individuals within the same species, since most generalist predators are cannibals [3]. Cannibalism occurs very frequently in nature and has been documented in more than 1300 species [4,5]. For many arthropods, cannibalism is a normal phenomenon, not an anomaly. Cannibalism has been documented in many insect orders, including Odonata, Orthoptera, Thysanoptera, Hemiptera, Trichoptera, Lepidoptera, Diptera, Neuroptera, Coleoptera and Hymenoptera. It occurs among predatory species and herbivores, involving predation by the mobile adults and larvae or nymphs on each other, and on immobile eggs and pupae [5,6]. There are many types of cannibalism, e.g., filial cannibalism as an energetic benefit [7]; sibling cannibalism [8,9] and intrauterine cannibalism in parasitoid insects [10], in which the cannibalism can increase the survival rate when food is scarce [11]; sexual cannibalism, in which a female insect cannibalizes her male mate during copulation [12]; cannibalism as competition [13]; or parasitizing offspring [5], etc.

In many systems, IGP and cannibalism occur together [14], and IGP is often associated with cannibalism [15,16]. Omnivory can be viewed as a strategy to reduce intraguild predation levels (and cannibalism) as it may allow omnivores to change locations and feed on plants under threat of predation [1].

From a practical point of view, the effects of IGP and cannibalism in biological pest control have received unequal attention. Many previous studies have been dedicated to the effects of IGP on the efficacy of natural enemies [17]. Most frequently, IGP is reported to be damaging or antagonistic [16,17,18,19,20,21,22] although it may sometimes have a neutral [23] or beneficial (synergistic) effect [24,25].The effect of cannibalism has received less modelling attention, which is curious since it is often associated with IGP. Moreover, it is an important impediment to efficiency in the mass production of biological control agents [26,27,28,29,30,31]. In addition, in augmentative biological control, releases can result in high densities of natural enemies at low pest levels, or before the pest appears on the crop [32]. Thus, cannibalism could exert an important influence on biological control outcomes [33,34,35,36]. Finally, because cannibalism is ubiquitous in food webs and frequent in systems where predator and prey share a common resource (IGP), its impacts on interspecific interactions and community dynamics and structure need to be better understood [37,38].

Biological pest control systems are very complex, especially in greenhouse crops where various beneficial organisms may be employed at the same time (predators, parasitoids and entomopathogens) in the same crop cycle to control different phytophagous species [32]. In such systems, it becomes more important, and sometimes fundamental, to recognize all the ecological relationships because the success of the system may depend on this knowledge [25]. Information is limited on the effects of cannibalism regarding the efficacy of biological pest control in these systems when, for example, high densities of natural enemies are released in augmentative biological programs [39] or when the pest population is not present, or present only at a low density. At the same time, agro-ecosystems are usually modified to be simpler than natural ecosystems [40], involving fewer factors and fewer interactions, which could facilitate the interpretation of ecological relationships.

*Nesidiocoris tenuis* (Reuter) (Hem.: Miridae), an omnivorous species [41], was introduced into Europe [42,43] from an originally paleotropical distribution. The species feeds both phytophagously and zoophagously, and has been considered a crop pest [44,45]. Its prey range includes aphids, whitefly and eggs and larvae of small lepidopterans [46,47,48,49]. Conversely, *Nabis pseudoferus* Remane (Hem.: Nabidae) can be considered a generalist predator (a non-omnivorous species) [50]. The majority of the Nabidae studied also practice plant feeding but they are not able to develop in the absence of prey [51,52,53]. Plant feedings are believed to be for the purpose of water acquisition [54] and do little or no damage to the plant. This practice seems to help the predator to survive during prey scarcity [53]. *N. tenuis* is currently used as a biological control agent in tomato greenhouses to control the whitefly *Bemisia tabaci* (Gennadius) (Hem.: Aleyrodidae) and the tomato leaf miner *Tuta absoluta* (Meyrick) (Lep.: Gelechiidae) [32,39].

The other species, *N. pseudoferus* (Hem.: Nabidae), has a wide prey range and is considered to be an important predator of aphids [55,56], but also a voracious predator of lepidopterans and other groups of arthropods, including hemipterans and spider mites [57,58,59,60]. *N. pseudoferus* is also currently used as a biological pest control agent of lepidopterans in greenhouses crops [16,32].

According to the above and the types of omnivory mentioned above, *N. tenuis* is a “true omnivore” and *N. pseudoferus* is a “generalist predator”.

The aim of this work was to study the importance of cannibalism in two species of predatory bugs with different feeding behavior that are often used in biological control programs. The cannibalism performed by each species was studied, both in the presence and absence of prey, in relation to their ontogeny under laboratory conditions, after which the IGP between both species was assayed under similar conditions. Cannibalism by the generalist predator was also studied under microcosm conditions.

## 2. Materials and Methods

### 2.1. Ethics Statement

The *N. pseudoferus* specimens were collected using a sweep net from alfalfa grown on private land after obtaining the owner’s permission. The sampling methods, the collection of the experimental *N. pseudoferus*, the rearing under controlled conditions and the design and development of the experiments, etc.—for this species and the other insect species used in this work—agree with the Spanish and European legislation on the protection of animals used for scientific purposes, which exclude invertebrates/insects.

### 2.2. Biological Material

A colony of *N. pseudoferus* was established from a population collected in Pinos Puente (37.248258° N, 3.765974° W) and Atarfe (37.218402° N, 3.713381° W), Granada, Spain, and reared under laboratory conditions for 25 generations (3 years; 1.5 months/generation) before being used in the experiments. Every year new individuals collected at the same locations were added to the laboratory population to avoid inbreeding and loss of genetic variability. A colony of *N. tenuis* was established from material purchased from a commercial producer (Nesidiocontrol^®^, Agrobio S.L., La Mojonera, Almeria, Spain) and reared in the lab for two generations before being used in the assays. Frozen eggs of *Ephestia kuehniella* Zeller (Lep.: Pyralidae), which were also used in the experiments, were purchased from a commercial supplier (Ephescontrol^®^, Agrobio S.L., La Mojonera, Almeria, Spain) and stored at −40 °C until use.

### 2.3. Laboratory Trials

Four laboratory assays, adapted from the methodology of Walzer and Shausberger [61] and Schausberger and Croft [62], were conducted under the physical conditions of 25 ± 1 °C, 60–80% relative humidity (RH) and a 16 h:8 h light:dark photoperiod.

#### 2.3.1. Experimental Design and Procedures

Seven-day-old adult females collected from the stock colony were assumed to be sexually mature and mated. Newly molted nymphal stages with hardened exoskeletons were selected to avoid presenting conspecifics during a vulnerable period of ecdysis [5]. All the individuals were isolated in plastic containers (500 mL) with a sponge (2.0 cm × 2.0 cm × 0.2 cm) moistened with distilled water and starved for 24 h prior to use in the assays. Individuals were then transferred to a new container in pairs, depending on the treatments described below, without refuge, water or food. The trials were performed over three days.

The treatments were as follows: (a) Cannibalism assay for *N. pseudoferus*: All 21 pairwise mathematical combinations, with repetitions and non-order from the following life stage/instar: adult female, V-, IV-, III-, II- and I-instar nymph (Table A1); (b) Cannibalism assay for *N. tenuis*: The treatments consisted of the same 21 combinations as above (Table A1); (c) *N. pseudoferus*–*N. tenuis* IGP-assay 1: In the hypergeometric distribution (in which selections are made from two subgroups without replacing members of the subgroup; this distribution differs from the binomial distribution in the lack of replacements) [63]: Subgroup 1 (6 elements): Adult female, V-, IV-, III-, II- and I-instar nymphs of *N. pseudoferus*, and Subgroup 2 (6 elements): adult female, V-, IV-, III-, II- and I-instars of *N. tenuis*, twenty-one pairs were chosen, as shown in Table A2, in which the two species were at the same or lower stage class; and (d) *N. pseudoferus*-*N. tenuis* IGP-assay 2: In the hypergeometric distribution in which there are two subgroups) that do not have elements in common: Subgroup 1 (5 elements): V-, IV-, III-, II- and I-instar nymphs of *N. pseudoferus*, and Subgroup 2 (5 elements): Adult female, V-, IV-, III- and II-instars of *N. tenuis*. Fifteen pairs were chosen, as shown in Table A3, in which *N. pseudoferus* was always in a lower stage class than *N. tenuis*.

Twenty repetitions were performed for each assay and treatment. All the assays were conducted identically on different days until all the treatments and repetitions were completed. We used the instantaneous sampling method [64,65] to analyze the survival times of the individuals. Each container was observed for one minute (the sample point) every 30 min (the sample interval) until the sixth hour of the first day. If no individuals died, the procedure was performed again on the second day, and if there was still no reaction, the procedure was repeated on the third day.

Additionally, 20 adult females and 20 nymphs from each developmental stage (instars) were selected from the rearing populations, placed under the same conditions as before, mounted in alcohol and measured (length and width) using a micrometer under a binocular microscope.

#### 2.3.2. Statistical Analysis

The cumulative survival times of the nymphal instars or adults, caged with either a conspecific or heterospecific, were analyzed using the Kaplan–Meier procedure [66,67]. This procedure is a method of estimating time-to-event models in the presence of censored cases. Within the Kaplan–Meier procedure, the equality of survival functions was compared with Breslow tests [68] using IBM SPSS version 25 software [69].

The mortality data were expressed as percentages, and survival times in hours. The data obtained in the cannibalism assay of *N. pseudoferus* and *N. tenuis*, as well as the values corresponding to IGP *N. pseudoferus*–*N. tenuis*, were adjusted to follow the non-linear (quadratic) regression:

(1) $$Y=a+b\cdot x+c\cdot x^{2}\;$$

where *Y* = the mortality or survival time and *x* is the size ratio (the difference of the product of length x width of the predator minus the product of length x width of the prey), expressed in mm^2^. The size difference parameter was used because, for many species, cannibalism and IGP are more related to size disparity than to absolute size [5]. The previous equation was adjusted by non-linear regression using the Statgraphics Centurion version 18 statistical software package [70].

### 2.4. Microcosm Trials

Regarding the high level of *N. pseudoferus* cannibalism found in previous trials, the starting hypothesis was to check whether the presence of alternative prey and refuge could significantly reduce such cannibalism. To test this hypothesis, two trials were conducted under microcosm conditions to evaluate filial and sibling cannibalism as a function of the predator developmental stage and prey density.

#### 2.4.1. Experimental Design and Procedures

The two trials were performed with individuals selected from the lab stock colony of *N. pseudoferus*, using the same procedure as described above. Individuals were isolated in 500 mL plastic containers (as above) and starved for 24 h prior to the assays, after which the individuals were transferred to a new plastic container (40.0 cm × 30.0 cm × 21.0 cm; used as a microcosm). The containers had two holes on the top (5.0 cm in diameter) covered with mesh. One tomato plant, cv Vernal^®^, Enza Zadem (ca. 23 cm high, with 7–8 leaves), was included in each container. *E. kuehniella* eggs were used as prey and they were always provided in the same way to avoid prey search problems, and conflicts between conspecifics, as well as to ensure uniform distribution, as described below. The *E. kuehniella* eggs were adhered with water to a 15 cm-long portion of sisal rope. To ensure the correct prey weight, all the ropes were weighed with precision scales before and after the trial. The rope pieces with the *E. kuehniella* eggs attached were then entwined around the plant stem to eliminate any predator in one location. *Nabis* adults are known to prefer the upper parts of the plant (inside the plant canopy) while the immature stages tend to stay lower down on the plant, outside the canopy [71,72].

The factorial design used a single factor at two levels: (1) the presence or absence of adult females, and (2) the *E. kuehniella* prey density. Each treatment was repeated four times.

In the first trial, 10 I-instar nymphs were placed in each container, whereas in the second assay, there were five III-instar nymphs per container. The same prey densities were used as in the first assay: 0, 0.006, 0.011 and 0.040 g/day; and in the second assay: 0, 0.011, 0.040 and 0.080 g/day.

Both trials were performed at 25 ± 2 °C, 60–80% RH and a 16:8 L:D photoperiod. The containers were examined daily for *E. kuehniella* prey replenishment, and the developmental stage of the predators was checked. The first assay was terminated when 50% of the nymphs molted to the III-instar. Similarly, the second assay ended when 50% of the nymphs reached the adult stage. The number of individuals surviving to the end of the assay was then recorded. In addition, the females used in the second microcosm assay were previously marked [73]: A dot of 0.4 pigment liner (art. no.: 308 04-9, Staedtler^®^, Nuremberg, Germany) was applied to each quadrant of the pronotum.

#### 2.4.2. Statistical Analysis

The survival percentages were subjected to a generalized linear model (GZLM) analysis using IBM SPSS version 25 software [69]. The models were fitted by maximum quasi-likelihood estimation using the GenLin procedure with normal errors and the identity function. In each trial, the significance of the model was assessed with an Omnibus test (to test whether the explained variance in a data set is significantly greater overall than the unexplained variance). For each regression effect specified in the model, a Wald statistical test was carried out, which is based on the linearly independent pairwise comparisons among the estimated marginal means. Then, the mean values were compared pairwise, with significance indicated at *p* = 0.05.

To order to estimate the nymph mortality specifically due to cannibalism by adult females, the Henderson–Tilton equation [74] was applied:

(2) $$M_{C}=\frac{M_{t}-M_{t}^{\prime }}{100-M_{t}^{\prime }}*100\;$$

where *M_C_* is the corrected percentage of mortality due to adult females, *M_t_* is the percentage of nymphal mortality in the presence of adult females at the end of the assay and *M’_t_* is the percentage of nymphal mortality in the absence of adult females at the end of the assay.

## 3. Results

### 3.1. Stage Structure

**Figure 1:**
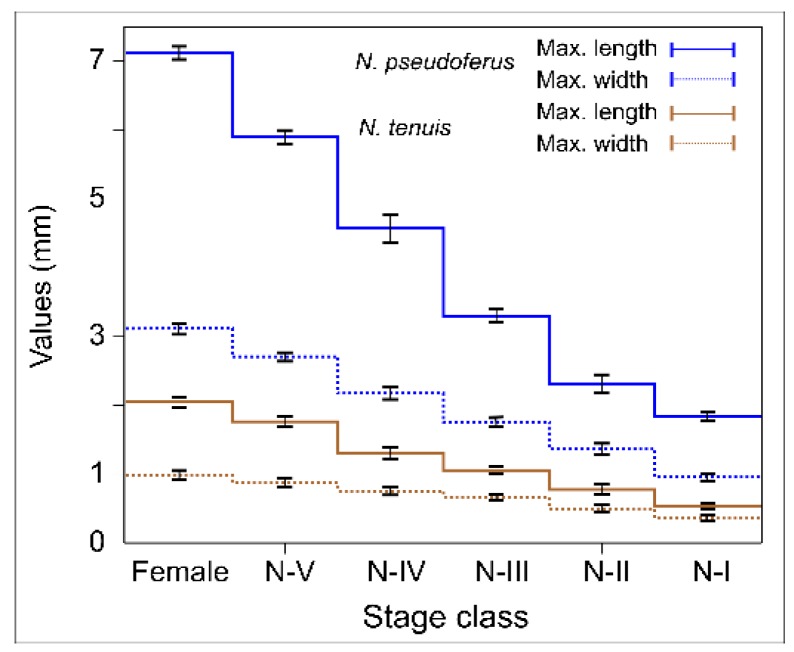
Average maximum lengths and widths (mm ± SE) of *Nabis pseudoferus* and *Nesidiocoris tenuis* according to their post-embryonic development.

The size of *N. pseudoferus*, especially the length, increased from 1.84 ± 0.04 mm for the first instars to 7.11 ± 0.06 mm in adult females, while in *N. tenuis*, it increased from 0.96 ± 0.03 mm to 3.12 ± 0.02 mm (Figure 1). In contrast, only the last nymph instars (IV- and I-instars) and adult females of *N. tenuis* had the same or greater size than the first nymphal instars of *N. pseudoferus* (I- and II-instars).

### 3.2. Laboratory Trial 1: N. pseudoferus Cannibalism

**Figure 2:**
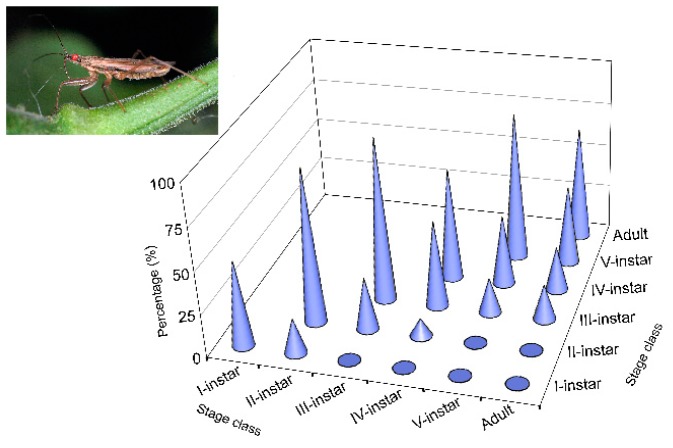
Survival (%) from *Nabis pseudoferus* cannibalism by life stage when caged with other conspecifics of the same or lower stage class, over 72 h, under laboratory conditions (25 ± 1 °C and 60–80% RH) without prey.

Cannibalism by *N. pseudoferus* in the absence of prey was very high in all nymphal instars and adult females (Figure 2). The average survival of all the bugs was quite low (41.7%). Higher values were only observed when both conspecifics were in the same developmental stage. Average survival increased from 11.7% for first instars to 65.0% for adult females.

The Kaplan–Meier procedure revealed significant differences within the treatments (Breslow test, generalized Wilcoxon *χ*^2^ = 68.925, df = 5, *p* < 0.0001) (Table A4). Except for one case, the differences in the survival time were all significant, indicating high levels of cannibalism, but with somewhat different values. For all stages, the average survival time was 38.05 ± 1.38 h, just over half the experimental time limit (72 h). The survival time increased from 23.17 ± 2.02 h for the I-instars to 63.00 ± 2.91 h for adult females. The highest survival times were observed when conspecifics from the same developmental stage were paired. Lower survival times were observed when first and second instar nymphs were paired with later developmental stages.

**Figure A1:**
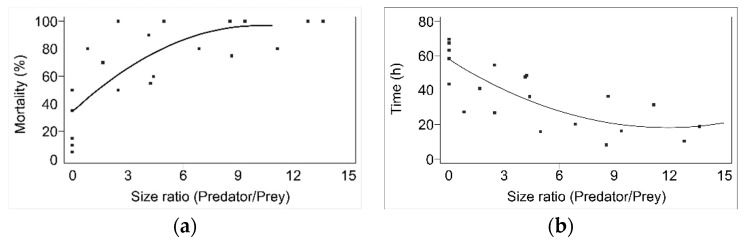
Relationship between mortality (%) (**a**) and survival time (h) (**b**) of *Nabis pseudoferus* as a function of the size ratio between cannibal and prey.

The mortality and survival time adjusted for the size ratio are shown in Figure A1a,b. The values for the *a*, *b* and *c* parameters were 34.32 ± 7.51, 12.29 ± 3.36 and −0.60 ± 0.27, and 57.75 ± 4.66, −6.70 ± 2.08 and 0.28 ± 0.17 for the mortality and survival time, respectively. Both models were highly significant (*F* = 15.03, df = 2, *p* = 0.001; and *F* = 15.32, df = 2, *p* = 0.001, respectively). One could observe that prey mortality (in smaller sizes) increased with an increasing size difference between the conspecifics. In other words, the survival time decreased with an increasing size disparity.

### 3.3. Laboratory Trial 2: N. tenuis Cannibalism

**Figure 3:**
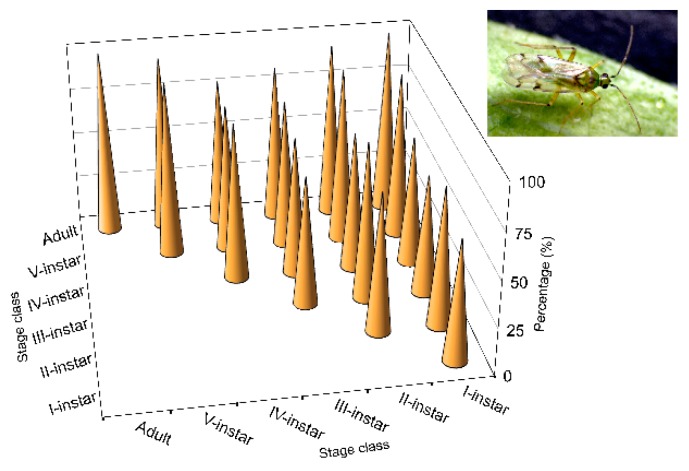
Survival (%) from *Nesidiocoris tenuis* cannibalism by life stage when caged with other conspecific of the same or lower stage class, over 72 h, under laboratory conditions (25 ± 1 °C and 60–80% RH) without prey.

In contrast to *N. pseudoferus*, *N. tenuis* showed a lower level of cannibalism (Figure 3). The average survival time for all stages was 82.6%, almost double that of *N. pseudoferus*. The survival percentage was lower for the I-instars (61.0%) and it increased up to 100% for adult females. The lowest survival percentages were lower than the average of the I- to III-instar nymphs from the same developmental stages (Figure 3). Additionally, unlike the other species, the most developed nymphal stages and the adult females exhibited little or no more cannibalism than the first instars.

The aggressiveness of *N. tenuis* in relation to its conspecifics, measured as survival time (Table A5), was also very low, with an average value of 63.19 ± 0.95 h over the 72 h trial time. Despite the overall comparison in the Kaplan–Meier procedure, significant differences between treatments were found (Breslow test, generalized Wilcoxon *χ*^2^ = 11.443, df = 5, *p* < 0.043) for the adult females by comparing the strata or pair-only differences. The data found in the *N. tenuis* trial in the absence of prey or a food source (e.g., the plant) demonstrated very low cannibalistic behavior.

**Figure A2:**
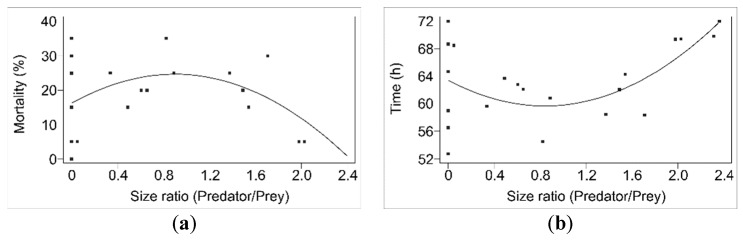
Relationship between mortality (%) (**a**) and survival time (h) (**b**) of *Nesidiocoris tenuis* as a function of the size ratio between cannibal and prey.

Figure A2a,b shows the nonlinear adjustments of mortality and victim survival in response to *N. tenuis* cannibalism. The values for the *a*, *b* and *c* parameters were 16.16 ± 3.53, 18.89 ± 9.16 and −10.54 ± 4.15, and 63.42 ± 1.77, −8.85 ± 4.57 and 5.26 ± 2.07, respectively. In this case, the model’s adjustments to mortality and survival time were significant (*F* = 4.16, df = 2, *p* = 0.0328; and *F* = 4.87, df = 2, *p* = 0.0204, respectively).

### 3.4. Laboratory Trial 3: IGP N. pseudoferus–N. tenuis Assay 1

From the overall comparison in the Kaplan–Meier procedure, it was determined that there were significant differences between treatments (Breslow test, generalized Wilcoxon *χ*^2^ = 74.582, df = 5, *p* < 0.0001).

Table A6 shows the aggressiveness of *N. pseudoferus* when paired with *N. tenuis* individuals at the same developmental stage. The average survival time values were very short compared with those observed for *N. pseudoferus* in the cannibalism assay (Table A4). The average time of the trials was 16.04 ± 0.60 h, which was less than one-quarter of the exposure time (72 h). The low survivorship time of *N. tenuis* adults in the presence of *N. pseudoferus* adult females was notable (Table A6), with a value of 3.53 ± 1.43 h, which was very significant compared to the other values. This might be because the higher prey mobility (due to wings) encourages more intensive predation by *N. pseudoferus* females. However, the survival time for *N. tenuis* I-instars seems to be very similar to that observed for *N. pseudoferus* I-instars (Table A4), indicating that adult females of both species were equally aggressive toward first instars.

The failure of any *N. tenuis* stages to survive 72 h indicates that *N. tenuis* is prey for *N. pseudoferus*. There was no mortality of any *N. pseudoferus* stage as a result of *N. tenuis* predation. Thus, another IGP trial was carried out, as described in the following section, to evaluate the IGP when *N. pseudoferus* was always in a lower stage class than *N. tenuis*.

### 3.5. Laboratory Trial 4: IGP N. pseudoferus–N. tenuis Assay 2

Due to the very low survival of *N. tenuis* as IGP-prey in relation to the actions of *N. pseudoferus* as an IGP-predator (as indicated in the previous section), a second trial was carried out in which the size differences between the two species were smaller. There were significant result differences between treatments (Breslow test, generalized Wilcoxon *χ*^2^ = 280.776, df = 1, *p* < 0.0001) for the overall comparison in the Kaplan–Meier procedure (Table A7). The mean survival time of the different *N. pseudoferus* stages was 68.76 ± 1.15 h compared to 35.23 ± 0.74 h for *N. tenuis*. This last value is much higher that found for this species in the previous trial.

**Figure 4:**
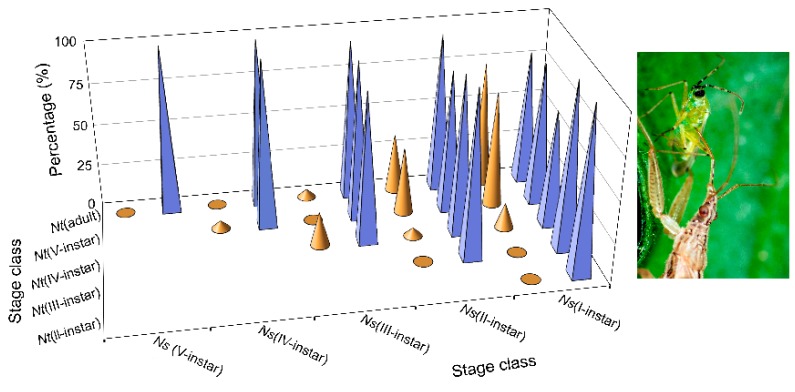
Stage-specific survival (%) of *Nesidiocoris tenuis* (brown cone, Nt) and *Nabis pseudoferus* (blue pyramid, Ns) in intraguild competition (IGP) when caged singly with the other species for 72 h under laboratory conditions (25 ± 1 °C and 60–80% RH) without prey, and when *N. pseudoferus* was always in a lower stage class than *N. tenuis*.

The survival of *N. pseudoferus* in the V- and IV-instars was 100%, but it was slightly lower in the earlier stages (70–100%) (Figure 4). Conversely, the survival of *N. tenuis* was low (Figure 4), but higher than in the previous trial, in which no individuals survived to the end of the assay. The results as a whole lead us to say that, in terms of IGP, size differences are very important, as indicated in the cannibalism trials.

It has to be pointed out that *N. pseudoferus* I-instar aggressiveness towards *N. tenuis* II-instars (19.38 ± 1.81 h) (Table A7) is similar to that of *N. pseudoferus* I-instars towards *N. tenuis* I-instars (20.60 ± 1.60 h) (Table A3), although both of the values were substantially lower than that found in I-instar cannibalism for *N. pseudoferus* (43.60 ± 6.58 h) (Table A4). This seems to indicate the cannibalistic intensity, which was lower in this stage than in the non-conspecific.

**Figure A3:**
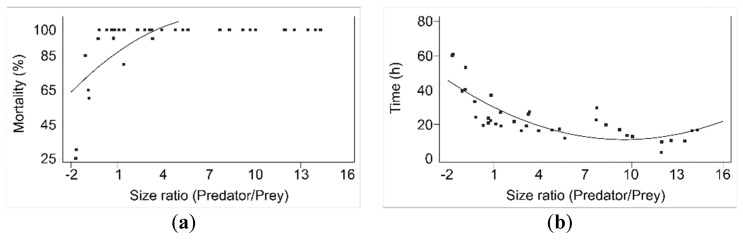
Relationship between mortality (%) (**a**) and survival time (h) (**b**) in the predation of *Nesidiocoris tenuis* by *Nabis pseudoferus*, depending on the size ratios between prey-predator, when *N. pseudoferus* was always in a lower stage class than *N. tenuis*.

The model adjusted to the *N. tenuis* mortality percentage and survival time, as the data of the two former assays (IGP), is shown in Figure A3a,b. At the same time, the parameter values *a*, *b* and *c* were 80.04 ± 3.37, 7.25 ± 1.47 and −0.46 ± 0.12, and 34.61 ± 2.04, −4.95 ± 0.90 and 0.26 ± 0.07 for the mortality and the survival time, respectively. The same was true for *N. pseudoferus* cannibalism, as the IG predation produced an increase in mortality and a decrease in the survival time for the IG prey, with an increase in the size differences between them in both cases.

### 3.6. Microcosm Trials: Effects of the Prey Density

#### 3.6.1. *N. pseudoferus* Cannibalism in the I- to III-Instars

**Figure 5:**
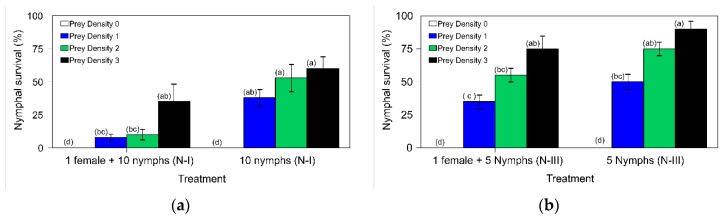
Survival (%) (±SE) of the *Nabis pseudoferus* instars: (**a**) N-I to N-III) or (**b**) N-III to adult in the presence or absence of an adult female, according to the prey density. The microcosm trial was performed using tomato plants under laboratory conditions (25 ± 2 °C and 60–80% RH). In each figure: the values with different superscript letters (a, b, c, and d) in a column are significantly different (*p* < 0.05).

Figure 5a shows the survival percentage for the *N. pseudoferus* nymphs (I- to III-instars), depending on prey density, and the presence or absence of adult females. The GZLM analysis showed that the model was highly significant (likelihood ratio *χ*^2^ = 45.431, df = 7, *p* < 0.0001). The presence of adult females (likelihood ratio *χ*^2^ = 20.998, df = 1, *p* < 0.0001), prey density (likelihood ratio *χ*^2^ = 33.356, df = 3, *p* < 0.0001) and interactions (likelihood ratio *χ*^2^ = 10.134, df = 3, *p* = 0.0170) had significant effects on nymphal survival. Survival was zero in the absence of prey and increased with prey density. Similarly, the survival of nymphs was lower in the presence of adult females than in their absence for prey densities 1, 2 and 3, respectively (Figure 5a). However, there were no significant differences in nymphal survival at densities 1, 2 and 3 in the absence of adult females, nor in the presence of adult females at high prey densities (Figure 5a).

#### 3.6.2. *N. pseudoferus* Cannibalism in the III-Instar to Adult Stages

For nymphs in a more advanced developmental stage (III-instar to adult), the GZLM analysis showed the model was highly significant (likelihood ratio *χ*^2^ =80.823, df = 7, *p* < 0.0001). The presence of adult females (likelihood ratio *χ*^2^ = 11.811, df = 1, *p* < 0.001) and prey density (likelihood ratio *χ*^2^ = 79.230, df = 3, *p* < 0.0001) had significant effects on survival, with no significant interaction between both factors (likelihood ratio *χ*^2^ = 10.134, df = 3, *p* = 0.190).

The nymphal survival for prey densities 1, 2 and 3 (the III-instar to adult trial) in the absence of adult females was 50.0% ± 5.8%, 75.0% ± 5.0% and 90.0% ± 5.8%, respectively, and this was lower in the presence of females, 35.0% ± 5.0%, 55.0% ± 5.0% and 75.0% ± 9.6%, respectively (Figure 5b). There were no significant differences in nymphal survival for each prey dose evaluated (1, 2 and 3) in the presence or absence of adult females. Therefore, compared to the previous trials, these values seem to indicate that, at the initial developmental stages of *N. pseudoferus* (the I- to III-instar trial) in the presence of different prey doses, there is a higher incidence of adult female cannibalism than in the later nymphal developmental stages, as shown in the previous cannibalism trials. Using the Henderson–Tilton equation, the mortality values for adult female cannibalism were 79.0%, 81.1% and 41.7% in the first microcosm assay, and 30.0%, 26.7% and 16.7% in the second, for prey densities 1, 2 and 3, respectively. Thus, in the presence of plant and prey, most cannibalism is carried out by adult females.

## 4. Discussion

The predatory species *N. pseudoferus*, which feeds on food sources from more than one trophic level, may be considered “trophic omnivorous”, according to Coll and Guershon [1]. It is considered a generalist predator; however, with regard to the diversity of taxonomical groups attacked, it is more specialist than other generalist predators, for instance, spiders, which are able to feed on several trophic levels [50]. *N. pseudoferus* was strongly cannibalistic when prey was absent. Individuals in the later developmental stages performed more acts of cannibalism, especially adult females. The results are within the general rule for cannibalism [5]. In contrast, the cannibalism rate for the omnivorous species *N. tenuis* was substantially lower; this is a “true omnivore”, following the terminology of Coll and Guershon [1], a particular case of trophic omnivory in which the consumer feeds on both plants and prey. For this species, the same nymphal instars (I, II and, to a lesser extent, III) were cannibalized by conspecifics of the same developmental stage (sibling cannibalism) (Figure 3 and Table A4). All of this serves to differentiate the two species. It also means that cannibalism in *N. tenuis* is an exception to the general rule of cannibalism, in which the largest (and older) individuals commit more acts of cannibalism than the smaller (and younger) individuals. This is similar to other exceptions cited in other species, such as certain species of fish, dragonfly larvae and parasitoid larvae that are more cannibalistic when smaller (younger) [5].

There are few studies published studies on cannibalism in *Nabis* species, with the exception of the studies performed on the American species *N. alternatus* Parshley [75,76], as well as on the European species *Himacerus apterus* F. [77]. The observed results regarding the incidence of cannibalism in *N. pseudoferus* are larger than those cited for *N. alternatus*.

On the other hand, the cannibalism rate for *N. pseudoferus* in the absence of prey is comparable to those cited in spiderlings of several wolf spider species [4,78,79]. However, in other species of this spider group, the cannibalism rate is lower [80,81].

In relation to other arthropod groups, the values found for *N. pseudoferus* were similar to those cited for larvae preyed upon by adult females in some species of predatory mites (Acari: Phytoseiidae) [62].

Cannibalism in *N. pseudoferus*, as commented on before, is very important and seems to be closely related to the absence of prey, as well as to size differences between the victim and the predator, as can be observed in the mortality percentages (Figure A1a) and survival times for the different developmental stages studied (Figure A1b). The importance of size differences in cannibalism has been cited and widely documented for scorpions [5,82], spiders [8,79,82,83] and predatory coccinellids [84,85], as well as for other invertebrate and vertebrate species [86,87]; however, it has seldom been studied in insects, with the exception of the work by Laycock et al. [88].

Analyzing the importance of size differences in *N. pseudoferus* cannibalism in more detail, for some species (e.g., fish or wolf spiders), it was reported that there is a predator–prey size difference threshold at which cannibalism takes place [5,83]; this does not seems to be the case in *N. pseudoferus*. However, only the papers by Polis [5,86] have studied size and cannibalism in detail. Polis [86] found that the relationship between size (size ratio: larger/smaller) and cannibalism was linear in desert scorpions. However, for *N. pseudoferus*, the relationship between size and cannibalism, whether the mortality percentage or the survival time, are not linear. The differences could be due, at least in part, to differences in development and life-cycle duration, as in the scorpion species studied, e.g., *Paruroctonus mesaensis* (Stahnke), which has a life cycle > 60 months compared to the short developmental period of *N. pseudoferus* (30 days; unpublished data), and in part due to different predation behavior.

A nonlinear relationship between the mortality, or the survival time, and the size ratio was found in *N. pseudoferus* (Figure A1a,b), which seems to be because of two effects: size differences and developmental stages, given that size differences are fundamental for cannibalism to occur. However, cannibalism is also influenced by the developmental stage of the predator, as can be observed from the survival percentages and survival times (Figure A1a,b). Therefore, cannibalism varies with the different developmental stages of *N. pseudoferus*, as measured in the survival percentage and the survival time.

The cannibalism results for *N. tenuis* show very low rates in the absence of prey or other food sources (e.g., plants), and in the absence of water. These results accord with those of Moreno-Ripoll et al. [89] for I- and II-instars of the same species in the absence of prey, although these authors reported different cannibalism results for adult females. In our work, there was no case of cannibalism between adult females whereas these authors did cite adult female cannibalism. The differences could be explained by the higher densities of female used by these authors. A higher density increases the number of encounters, and thus more cannibalism occurs [5,82]. Additionally, the survival values for *N. tenuis* in the presence of conspecifics are similar to those found in *Macrolophus pygmaeus* Wagner (Hem.: Miridae) [90].

Higher values of *N. tenuis* cannibalism for were observed (Figure 3) in the first three nymphal instars, performed by conspecifics at the same stages, as mentioned above. It should be noted that these *N. tenuis* nymphal instars have a higher degree of phytophagy. Thus, the I-instars of the species are able to survive until they become III-instars by feeding only on plant material [91,92,93]. In contrast to the III-instars, the species shows greater zoophagy [91]; this is contrary to the results that showed reduced cannibalism at that stage and at subsequent stages. Perhaps in the first three instars, cannibalism is not caused by the need to eliminate potential competitors, as cited for other species [4,5,14,85,94].

The results showing low levels of cannibalism in omnivorous *N. tenuis* suggest that omnivores sustain themselves on plant sources in the absence of prey without the need to resort to cannibalism, as stated by Leon-Beck and Coll [95]. Moreover, their potential as a phytophagous species, as observed in our results, is opposite to that reported by Bernays [96] for cannibalism in phytophagous insects, suggesting that in this group of insects, cannibalism is more common among generalist than specialist herbivores.

For *N. tenuis*, despite finding weak cannibalism, we also established a nonlinear relationship between the mortality percentage or survival time, and the size differences between the predator and the victim (Figure A2a,b); this contrasts with *N. pseudoferus*, where we found cannibalism differences occurring at low to intermediate sizes. This would suggest that cannibalism is more influenced by behavior, mainly of the early instars, than the size differences of the conspecifics.

In this work, the differences between *N. pseudoferus* and *N. tenuis* in the cannibalism rate and the attack stage, when both species share the same ecological niche [97], could be due to their different diets: *N. pseudoferus* is a “non-omnivorous predator” and *N. tenuis* is a “true omnivore”. Thus, in the absence of prey, *N. pseudoferus* cannot opt for any other food source than cannibalism whereas under the same circumstances, *N. tenuis* can choose to feed phytophagously. In contrast, omnivory could be a strategy to reduce IGP levels (and cannibalism) as it allows omnivores to change their location and to feed on plants in the presence of other predators [1].

From the IGP trial results, *N. pseudoferus* acts as an IG predator and kills *N. tenuis* as IG prey (Figure 4). It is an asymmetrical relationship in favor of the former species, depending on the size differences between the species (Figure 1). This confirms the work of Polis et al. [14], which states that relative body size and the degree of trophic specialization are the two most important factors influencing IGP frequency and direction. Most IGP occurs in systems with size-structured populations and is carried out by generalist predators who are usually larger than their intraguild prey. Many of these IG predators also cannibalize smaller conspecifics.

By studying *N. tenuis* predation at different developmental stages by *N. pseudoferus* in the two IGP tests performed, we can observe that both the survival percentage and the survival time are strongly influenced by size differences between the predator and prey (Figure A3a,b; Table A6 and Table A7)—this is known to happen in cannibalism, and has been shown, not only in our results, but also in numerous other studies [5,14,76,98,99].

However, in the present study, we found exceptions to the above relationship of size and IGP. In general, the different *N. tenuis* developmental states are smaller in size than those of *N. pseudoferus*, except for the adult stage of the *N. tenuis*, which is very similar to the III-instar state of *N. pseudoferus* (and larger than the I- and II-instars), whereas the V-instars of *N. tenuis* are larger than the I- and II-instars of *N. pseudoferus* (Figure 1). Despite these size differences, similar survival percentages and survival times were observed for *N. tenuis* adult females paired with I-instars of *N. pseudoferus* (Figure 3 and Table A7). In the other cases where there was an equal or smaller size, *N. pseudoferus* predated *N. tenuis*. This may be motivated (in the absence of molting individuals) by the fact that, for smaller or same-sized individuals, *N. pseudoferus* exhibits better-suited predatory behavior for capturing and killing than does *N. tenuis*. We know that *Nabis* species inject venom into their prey [100,101] and/or have better morphological adaptations (raptorial forelegs) [102] (Figure 2), characteristics that are not present in the other species. Such behavior in predatory *Nabis* species was observed when they were attacking larger, phytophagous species (e.g., *Spodoptera exigua* (Hübner), Lep.: Noctuidae) [103].

The importance of the presence of prey and refuge (the plant) in the *N. pseudoferus* cannibalism has been underscored in both microcosm assays (Figure 5a,b). This had already been cited in numerous studies on the presence and density of prey [9,34,84] and refuge [4,19]. However, in such circumstances, the filial cannibalism level of *N. pseudoferus* is still very high, especially by adult females on the first instars (I- to III-instars) (Figure 5a), more so than on the later stages (III to V-instars) (Figure 5b). This could explain the location of individuals within the plant. In the absence of other predatory species, the females lay eggs mostly into the leaf petioles spread out equally over the height of the plant [104]. Moreover, *Nabis* adults prefer to sit on the top or slightly lower in the plant canopy, while immature individuals are found lower down in the plant [71,72].

Insects and other arthropods, unlike vertebrate species, have complex life cycles in which the successive stages may differ more dramatically, both in physical appearance and in their ecological role [105,106,107,108]. The findings from the two species studied indicate that cannibalism depends not only on the species, but also on their stage structure. Most ecological models in contemporary ecological theory ignore the implications of the age and size variation, particularly within populations. This is also true for empirical studies, both experimental and non-experimental [38]. However, recent studies show that stage structure can modify the dynamics of consumer–resource communities owing to stage-related shifts in the nature and strength of the interactions that occur within and between populations [108]. Consequently, these results can help to develop mathematical models based on stage structure, by considering a more realistic species ontogeny. Furthermore, and from the applied standpoint, the results of this study also highlight the importance of cannibalism, and its repercussions, in current biological control systems.

## 5. Conclusions

1. The diet, whether strictly carnivorous or omnivorous, seems to have a marked effect on the cannibalism of the two species studied. This could be extended to other insect species.
2. Ontogenetic development in insects with a stage structure doubly influences the cannibalism and the intra-guild predation (IGP) by affecting both the individual prey and the predator.
3. The ratio of predator–prey size in relation to the rate of cannibalism and intra-guild predation (IGP) is not a linear relationship, as has already been pointed out in the literature on arthropods.
4. These findings can help to develop mathematical models based on stage structure, by more realistically considering species with this type of ontogeny.
5. From an applied standpoint, these study results also highlight the importance of cannibalism, and its repercussions, in current biological control systems for pest species.

## Appendix A

**Table A1:** Combinations used in cannibalism assays for *N. pseudoferus* and *N. tenuis* under laboratory conditions and without alternative prey.

Combinations					
Adult-Adult	**-**	**-**	**-**	**-**	-
Adult-NV	NV-NV	-	-	-	-
Adult-NIV	NV-NIV	NIV-NIV	-	-	-
Adult-NIII	NV-NIII	NV-NIII	NIII-NIII	-	-
Adult-NII	NV-NII	NV-NII	NIII-NII	NII-NII	-
Adult-NI	NV-NI	NV-NI	NIII-NI	NII-NI	NI-NI

NV, NIV, NIII, NII and NI: instars.

**Table A2:** Combinations used in the IGP *N. pseudoferus*–*N. tenuis*, assay 1, under laboratory conditions and without alternative prey, when both predatory species are in the same or lower stage class.

Combinations					
N.s.-N.t.	N.s.-N.t.	N.s.-N.t.	N.s.-N.t.	N.s.-N.t.	N.s.-N.t.
Adult-Adult	-	-	-	-	-
Adult-NV	NV-NV	-	-	-	-
Adult-NIV	NV-NIV	NIV-NIV	-	-	-
Adult-NIII	NV-NIII	NV-NIII	NIII-NIII	-	-
Adult-NII	NV-NII	NV-NII	NIII-NII	NII-NII	-
Adult-NI	NV-NI	NV-NI	NIII-NI	NII-NI	NI-NI

NV, NIV, NIII, NII and NI: instars. N.s. = *Nabis pseudoferus*, N.t. = *Nesidiocoris tenuis*.

**Table A3:** Combinations used in the IGP *N. pseudoferus*–*N. tenuis*, assay 2, under laboratory conditions and without alternative prey, when *N. pseudoferus* was always in a lower stage class than *N. tenuis*.

Combinations				
N.s.-N.t.	N.s.-N.t.	N.s.-N.t.	N.s.-N.t.	N.s.-N.t.
NI-Adult	NII-Adult	NIII-Adult	NIV-Adult	NV-Adult
NI-NV	NII-NV	NIII-NV	NIV-NV	-
NI-NIV	NII-NIV	NIII-NIV	-	-
NI-NIII	NII-NIII	-	-	-
NI-NII	-	-	-	-

NV, NIV, NIII, NII and NI: instars. N.s. = *Nabis pseudoferus*, N.t. = *Nesidiocoris tenuis*.

**Table A4:** Aggressiveness of *Nabis pseudoferus* cannibalism on different stage class, measured as survival time (hours) (±SE) when caged singly with other conspecifics for 72 h under laboratory conditions (25 ± 1 °C and 60–80% RH) and without prey.

Stage	Significance Level (*p*) (Breslow Test)														
	Adult		N-V		N-IV		N-III		N-II		N-I		χ^2^	df	*p* ^2^
	Value	*p* ^1^	Value	*p* ^1^	Value	*p* ^1^	Value	*p* ^1^	Value	*p* ^1^	Value	*p* ^1^			
**Adult**	63.0 ± 2.9	**─**	48.5 ± 5.7	0.065	36.5 ± 5.8	0.001	31.5 ± 5.6	0.0001	10.3 ± 2.5	0.0001	19.08 ± 2.4	0.0001	59.614	5	0.001
**N-V**			67.2 ± 2.8	─	36.2 ± 6.8	0.001	20.0 ± 6.2	0.0001	8.0 ± 2.3	0.0001	16.2 ± 2.1	0.0001	49.626	4	0.001
**N-IV**					58.3 ± 4.4	─	54.5 ± 4.3	0.6520	47.9 ± 3.6	0.0290	16.0 ± 2.7	0.0001	68.335	3	0.001
**N-III**							69.7 ± 2.3	─	41.01 ± 5.8	0.0001	26.9 ± 3.8	0.0001	30.974	2	0.001
**N-II**									67.5 ± 3.0	─	27.4 ± 5.7	0.0001	21.375	1	0.001
**N-I**											43.6 ± 6.6	─	─	─	─
**Average**	63.0 ± 2.9		57.8 ± 3.5		43.7 ± 3.6		43.9 ± 3.3		34.9 ± 2.8		23.2 ± 2.0	Total avg.		38.05 ± 1.38	

^1^ Significance level (*p*) of pairwise comparisons between high stage of development and the others (Breslow test). ^2^ Significance level (*p*) of Breslow test equality of survival distributions for different levels of stage factor.

**Table A5:** Aggressiveness of *Nesidiocoris tenuis* cannibalism on different stage class, measured as survival time (hours) (±SE) when caged singly with other conspecifics for 72 h under laboratory conditions (25 ± 1 °C and 60–80% RH) and without prey.

Stage	Significance Level (*p*) (Breslow Test)														
	Adult		N-V		N-IV		N-III		N-II		N-I		χ^2^	df	*p* ^2^
	Value	*p* ^1^	Value	*p* ^1^	Value	*p* ^1^	Value	*p* ^1^	Value	*p* ^1^	Value	*p* ^1^			
**Adult**	72.0 ± 0.0	─	68.5 ± 3.5	0.317	62.1 ± 4.5	0.037	64.3 ± 4.2	0.076	69.5 ± 2.5	1.000	72.0 ± 0.0	1.000	11.969	5	0.035
**N-V**			68.7 ± 3.3	─	62.8 ± 4.2	0.187	62.0 ± 4.5	0.187	69.4 ± 2.6	0.971	69.9 ± 3.5	0.594	3.537	4	0.472
**N-IV**					64.7 ± 4.0	─	60.9 ± 4.4	0.628	58.5 ± 5.4	0.285	58.4 ± 478	0.435	1.082	3	0.781
**N-III**							56.6 ± 5.5	─	63.7 ± 4.6	0.394	54.5 ± 5.5	0.431	2.510	2	0.285
**N-II**									59.0 ± 5.2	─	59.6 ± 4.9	0.789	0.720	1	0.789
**N-I**											52.8 ± 6.1	─	─	─	─
**Average**	72.0 ± 0.0		68.6 ± 2.4		63.2 ± 2.4		61.0 ± 2.3		64.0 ± 1.9		60.7 ± 1.9	Total avg.		63.2 ± 1.0	

^1^ Significance level (*p*) of pairwise comparisons between high stage of development and the others (Breslow test). ^2^ Significance level (*p*) of Breslow test equality of survival distributions for different levels of stage factor.

**Table A6:** Aggressiveness of *Nabis pseudoferus* predation (intraguild competition) on *Nesidiocoris tenuis* (both predatory species are in the same or lower stage class), measured as survival time (hours) (±SE), when caged one individual of each species during 72 h under laboratory conditions (25 ± 1 °C and 60–80% RH) and without prey.

Species/	Significance Level (*p*) (Breslow Test)														
Stage	*Nesidiocoris tenuis*														
*Nabis*	Adult		N-V		N-IV		N-III		N-II		N-I		*χ* ^2^	df	*p* ^2^
*pseudoferus*	Value	*p* ^1^	Value	*p* ^1^	Value	*p* ^1^	Value	*p* ^1^	Value	*p* ^1^	Value	*p* ^1^			
**Adult**	3.5 ± 1.4	─	9.7 ± 2.4	0.23	10.5 ± 2.8	0.0020	10.2 ± 2.2	0.0050	16.1 ± 2.2	0.0001	16.7 ± 2.0	0.0001	47.912	5	0.001
**N-V**			29.5 ± 3.7	─	19.6 ± 4.5	0.0570	16.7 ± 3.5	0.2190	13.4 ± 2.9	0.0040	12.9 ± 2.2	0.0001	12.282	4	0.015
**N-IV**					16.1 ± 3.5	─	16.7 ± 2.7	0.7040	17.2 ± 2.1	0.0580	11.8 ± 2.4	0.9460	5.560	3	0.135
**N-III**							21.6 ± 1.6	─	16.1 ± 2.1	0.0001	19.0 ± 1.9	0.0001	30.993	2	0.001
**N-II**									20.1 ± 1.8	─	19.0 ± 1.8	0.1440	2.137	1	0.144
**N-I**											20.6 ± 1.6	─	─	─	─
**Average**	3.5 ± 1.4		19.6 ± 2.7		15.4 ± 2.2		16.3 ± 1.4		16.6 ± 1.0		16.7 ± 0.9	Total avg.		16.0 ± 0.6	

^1^ Significance level (*p*) of pairwise comparisons between high stage of development and the others (Breslow test). ^2^ Significance level (*p*) of Breslow test equality of survival distributions for different levels of stage factor.

**Table A7:** Aggressiveness of *Nabis pseudoferus* predation (intraguild competition) on *Nesidiocoris tenuis* (*N. pseudoferus* was always in a lower stage class than *N. tenuis*), measured as survival time (hours) (±SE), when caged one individual of each species during 72 h under laboratory conditions (25 ± 1 °C and 60–80% RH) and without prey.

*N.t.*	Significance Level (*p*) (Breslow Test)																		
	Value		*p* ^1^	Value		*p* ^1^	Value		*p* ^1^	Value		*p* ^1^	Value		*p* ^1^	*χ* ^2^	df	*p* ^2^	*N.t.*
	*N. t.*	*N. s.*		*N. t.*	*N. s.*		*N. t.*	*N. s.*		*N. t.*	*N. s.*		*N. t.*	*N. s.*					
Stage	-	N-V		-	N-IV		-	N-III		-	N-II		-	N-I					Avg.
**Adult**	22.6 ± 1.0	72.0 ± 0.0	0.001	26.1 ± 2.7	72.0 ± 0.0	0.001	22.4 ± 3.1	68.5 ± 3.4	0.001	40.5 ± 5.3	69.6 ± 2.5	0.001	60.2 ± 4.6	60.2 ± 5.5	0.910	20.372	4	0.000	33.8 ± 2.2
**N-V**				27.1 ± 3.2	72.0 ± 0.0	0.001	36.9 ± 3.1	70.7 ± 1.3	0.001	53.2 ± 4.3	62.2 ± 4.8	0.670	60.9 ± 4.2	64.8 ± 4.3	0.300	12.446	3	0.000	45.3 ± 2.3
**N-IV**							27.1 ± 3.2	72.0 ± 0.0	0.001	33.3 ± 3.1	69.5 ± 2.5	0.001	39.2 ± 3.5	62.0 ± 4.5	0.000	2.745	2	0.250	37.0 ± 2.2
**N-III**										23.5 ± 2.0	72.0 ± 0.0	0.001	24.1 ± 0.5	72.0	0.001	1.516	1	0.210	23.8 ± 1.0
**N-II**													19.4 ± 1.8	72.0	0.001	─	─	─	19.4 ± 1.8
**Avg.**		72.0 ± 0.0			72.0 ± 0.0			69.6 ± 1.5			68.9 ± 1.4			66.2 ± 1.7					

^1^ Significance level (*p*) of pairwise comparisons between high stage of development and the others (Breslow test). ^2^ Significance level (*p*) of Breslow test equality of survival distributions for different levels of stage factor. *N.t.* = *Nesidiocoris tenuis*; *N.s.* = *Nabis pseudoferus*.
